# Should we adjust for delivery hospital in studies of air pollution and pregnancy outcomes?

**DOI:** 10.1097/EE9.0000000000000064

**Published:** 2019-09-12

**Authors:** David A. Savitz, Melissa N. Eliot, Kazuhiko Ito, Sarah Johnson, Justin Manjourides, Valery A. Danilack, Gregory A. Wellenius

**Affiliations:** Departments of a Epidemiology; b Obstetrics and Gynecology; c Pediatrics, Brown University, providence, Rhode Island; d New York City Department of Health and Mental Hygiene, New york; e Department of Health Sciences, Northeastern University Boston, Massachusetts.

**Keywords:** Air pollution, Confounding, delivery hospital, Pregnancy outcome

## Abstract

**Background:**

In conducting a study of ambient air pollution and pregnancy outcome in New York City, we identified delivery hospital as a potential confounder, given its association with both maternal residence and therefore air pollution exposure, and with clinical practices and as a potential marker of outcome misclassification in the coding of pregnancy complications. Motivated by evidence that adjustment for delivery hospital affected associations between air pollution and pregnancy outcome, we undertook a detailed empirical examination of the role of delivery hospital that warrants consideration by others addressing this topic.

**Methods:**

In a study of air pollution and pregnancy outcome, we identified births from 2008 to 2010 to residents of New York City and, after restrictions, included 238,960 in the analysis. Air pollution exposure estimates for ambient fine particles (PM_2.5_) and nitrogen dioxide (NO_2_) were derived from a community-wide exposure study and assigned based on geocoded maternal residence. We examined the impact of adjusting for delivery hospital and explored the relationship between delivery hospital and both exposure and pregnancy outcomes.

**Results:**

Statistical adjustment for delivery hospital markedly attenuated the relationship of air pollution with birth weight and gestational hypertension, with smaller effects on preterm birth and preeclampsia. Delivery hospital was associated with estimated maternal air pollution levels after adjusting for individual-level patient characteristics, more strongly for PM_2.5_ than for NO_2_. Delivery hospital predicted pregnancy outcome after adjustment for individual attributes, with larger hospitals and those that managed a greater volume of complicated cases having lower birth weight, more medically indicated preterm births, and more diagnosed gestational hypertension. Evaluation through the use of directed acyclic graphs illustrates the potential for adjustment for hospital to reduce residual spatial confounding, but also indicates the possibility of introducing bias through adjustment of a mediator.

**Conclusions:**

Based on these results, delivery hospital warrants closer consideration in studies of air pollution and other spatial factors in relation to pregnancy outcomes. The possibility of confounding by delivery hospital needs to be balanced with the risk of adjusting for a mediator of the air pollution—pregnancy outcome association in studies of this type.

## Introduction

An extensive and growing body of research suggests that there may be small adverse effects of ambient air pollution on risk of preterm birth, fetal growth restriction, and hypertensive disorders of pregnancy.^1–3^ Because the magnitudes of the observed associations are sometimes small, distinguishing between a causal effect of air pollution and even modest degrees of residual confounding is challenging. Location of maternal residence is often used as a marker of residential or community exposure to ambient air pollution, and this leads to well-justified concerns with spatial confounding by correlates of residential location such as socioeconomic status that are related to pregnancy outcomes.

Delivery hospital is a correlate of residential location that to our knowledge has not been previously considered as a potential confounder. Because women from a given neighborhood tend to deliver at nearby hospitals, average residential air pollution exposures are also likely to differ across women grouped by delivery hospitals, as effectively exploited in a series of air pollution assessments derived from multi-hospital clinical studies.^4,5^ Delivery hospital may also be independently predictive of pregnancy outcome through multiple pathways. In a substantial proportion of pregnancies, the timing of delivery is influenced in part by clinical decisions regarding the management of the pregnancy, given that nearly one-fourth of all births in the United States are the result of intervention in the form of labor induction or prelabor cesarean delivery.^6^ Consideration of intervening late in gestation is quite common, when the tradeoff between allowing the pregnancy to continue and delivering is most ambiguous. Such interventions directly affect the gestational age at delivery, with approximately 40% of all preterm births resulting from medical indication and intervention.^7^ Interventional delivery would also affect the birth weight distribution overall and at any given gestational age, with fetal growth as one of the key considerations leading to interventional delivery. Proclivity to intervene is known to vary over time, across individual clinicians, and by source of medical care^8^ and thus is likely to vary across delivery hospitals. Although these effects may be subtle, the air pollution effects of interest may also be of modest magnitude.

Pregnancy complications such as preeclampsia arise outside the delivery hospital and thus could not be affected by hospital practices. However, hospitals may differ from one another in their notation and coding of conditions in the discharge summary, particularly for conditions such as hypertensive disorders for which mild variants arising late in gestation are quite common. Coding of pregnancy complications is a manual process subject to individual judgments, which allows for the completeness and accuracy of notation and coding to vary across hospitals, potentially introducing varying degrees of misclassification.

Thus, delivery hospital is likely to be associated to some extent both with average residential levels of air pollution among expectant mothers and may influence the commonly studied outcomes of pregnancy, including preterm birth, birth weight, and hypertensive disorders. Given these potential associations, it is important to consider whether delivery hospital should be adjusted for as a potential source of confounding in examining the association between residential air pollution and pregnancy outcome or whether adjustment could introduce bias in assessing the causal effect of air pollution on outcome. A related concern is whether studies that rely on a single hospital or incomplete subset of hospitals (effectively conditioning on delivery hospital) may be vulnerable to selection bias through these associations.

We were motivated to investigate this issue by empirical findings across a series of studies on air pollution and pregnancy outcome in New York City and recognized that our own uncertainty regarding the appropriateness of adjusting for delivery hospital resulted in inconsistency across publications.^9–11^ Finding that adjustment for delivery hospital had a marked influence on some of the estimated associations between air pollutants and some pregnancy outcomes motivated us to undertake an empirical assessment of the phenomenon within our data. The large population, diversity of delivery hospitals, and unusually precise information on outdoor air pollution levels at the residential address provided an opportunity to more fully examine the influence of delivery hospital on the association between ambient fine particulate matter (PM_2.5_) and nitrogen dioxide (NO_2_) with preterm birth, birth weight, and hypertensive disorders of pregnancy.

## Methods

### Summary of data source and measures

Details of the data source and study methods are provided in previous publications.^9–11^ Briefly, we examined the associations between estimated residential levels of PM_2.5_ and NO_2_ and birth outcomes among New York City residents who gave birth in New York City hospitals between 2008 and 2010. Maternal exposures to PM_2.5_ and NO_2_ at residential address for each 2-week block of her gestational period were estimated using (1) the spatial variation derived from the New York City Community Air Survey data collected at 150 monitoring sites and a land-use regression model; and (2) the temporal variation derived from the US Environmental Protection Agency’s air quality network data. Details of the exposure assignment method, including validation, can be found elsewhere.^12^ Exposure estimates were assigned based on maternal residence at the time of delivery during the time period of the pregnancy, accounting for both spatial and temporal variation in air pollution levels. Starting with 348,585 births, we excluded patients with missing or implausible gestational age (<22 or >42 weeks), multiple gestations, congenital abnormalities, known smokers, missing or implausible birth weight (<500 or >5,000 g), missing covariates (e.g., education, parity), missing delivery hospital, or born in a hospital with fewer than 10 births over the course of the study period and restricted conception dates to avoid fixed-cohort bias, leaving 257,956 births in the data set with some additional restrictions for studying specific endpoints as noted below. Although most exclusions were made to avoid bias or refine the health outcome analyzed, we were unable to include 15,153 births (4.3%) with missing data on delivery hospital.

We considered several health outcomes based on birth records and hospital discharge data, all of which we have examined and described in greater detail in previous publications^9–11^: term birth weight in grams as a continuous measure (N = 238,960 after excluding preterm births), small-for-gestational age (SGA) defined as <10th percentile of weight for gestational age^13^ (N = 28,681 term births with SGA), preterm birth (<37 weeks’ gestation) (N = 18,996 preterm births), subsets of preterm birth identified as spontaneous (N = 13,719) or medically indicated (N = 5,277), and hypertensive disorders of pregnancy among 254,702 women without preexisting hypertension, classified as gestational hypertension (N = 5,340), mild preeclampsia (N = 6,368), or severe preeclampsia (N = 3,758).

The magnitude and direction of the impacts of hospital adjustment on odds ratios for air pollution exposures in the first and second trimesters were comparable in our published analysis of hypertensive disorders of pregnancy.^10^ Likewise, the impacts of hospital adjustment on odds ratios for air pollution exposures for the first and second trimesters in our analysis of preterm births were similar.^11^ We did not examine the hospital effects in our analysis of air pollution and birth weight,^9^ and, although birth weight reductions were observed for estimated exposures in all trimesters, the magnitude was greatest for the third-trimester exposures. Therefore, to narrow the scope of the analysis for examining the role of delivery hospital, we considered only one time window for each of the outcomes in these analyses, focusing on the one more likely to be of importance for the specific outcomes: first trimester exposure for hypertensive disorders of pregnancy, second trimester exposure for preterm birth, and third-trimester exposure for term birth weight.

### Data analysis methods

We used multiple linear regression for the analyses of term birth weight and multiple logistic regression for all other health outcomes. Associations are expressed as the change in birth weight or odds of the adverse outcome per 10 μg/m^3^ PM_2.5_ or per 10 ppb NO_2_. The initial model included adjustment for individual attributes of maternal age, ethnicity, education, Medicaid status, parity, prepregnancy body mass index (BMI), and conception year, as well as a score for socioeconomic status based on the census tract of residence^14^ adapted for New York City as described elsewhere.^9^ To examine the effect of additional adjustment for delivery hospital, we considered individual hospital as a fixed effect factor with 41 levels corresponding to the 41 hospitals included in the analysis. As a further sensitivity analysis, we also performed analyses considering hospital as a random (rather than fixed) effect in a linear mixed or generalized linear mixed model framework.

To gain further insights into the potential for confounding by delivery hospital, we examined the relation between delivery hospital and (1) estimated residential air pollution levels of women who delivered there and (2) the risk of adverse pregnancy outcomes. To quantify the relationship between hospital and residential air pollution levels of the women who delivered there, we estimated the variance accounted for in exposure estimates based on a linear regression model including the individual predictors and then added delivery hospital to the model to assess the incremental predictive value. To quantify the magnitude of the association between delivery hospital and maternal air pollution, we rank ordered the hospitals from lowest to highest average air pollution levels (adjusted for individual covariates), divided the distribution into quartiles, and calculated the difference in mean PM_2.5_ and NO_2_ comparing the first quartile (referent) to the second, third, and fourth quartiles of estimated exposure.

We followed a parallel strategy to assess the association between delivery hospital and pregnancy outcomes, with statistical methods appropriate for continuous and dichotomous outcomes. For birth weight, we assessed the variance accounted for by a linear regression model including adjustment for individual characteristics alone then a model that additionally adjusted for individual hospital. For the dichotomous outcome measures (preterm birth, pregnancy complications), we instead generated a pseudo-*R*^2^ using the McKelvey-Zavoina approach.^15^ Briefly, the McKelvey-Zavoina approach treats the dichotomous outcome as if it were a discretized continuous latent variable, and the pseudo-*R*^2^ value is defined as the proportion of the variance of that latent variable explained by a linear model of the predictors. With this indicator of variance accounted for, we followed the same steps of first examining a logistic regression model with individual predictors alone, then adding individual hospital to the model. To quantify the magnitude of association between hospital and pregnancy outcome, we rank ordered the hospitals by covariate-adjusted risk of the outcomes, divided the distribution into quartiles, and compared health outcomes in the upper three quartiles individually to the lowest quartile. This resulted in adjusted differences in birth weight and adjusted odds ratios for the other outcomes. We also developed a directed acyclic graph to aid our interpretation of results. All analyses were performed using R version 3.3.3 (R Foundation for Statistical Computing, Vienna, Austria). The protocol was reviewed and approved by the New York City Department of Health and Mental Hygiene Institutional Review Board.

## Results

We first empirically examined the impact of hospital adjustment on measures of association between air pollution and pregnancy outcome (Table 1). Compared to a model adjusting for a standard set of covariates, additional adjustment for delivery hospital as a fixed effect shifted measures of association closer to the null, except when the initial results were already essentially null. For PM_2.5_ and the indicators of fetal growth, the attenuation was substantial. For example, we found that a 10 μg/m^3^ increase in PM_2.5_ was associated with a 21 g (95% confidence interval [CI] = −30.3, −13.4 g) lower birth weight in the standard model, but a 9.8 g (95% CI = −19.1, −0.5 g) lower birth weight in a model additionally adjusted for delivery hospital. For preterm delivery, adjustment for hospital attenuated a small negative association with PM_2.5_ and to a lesser extent NO_2_. For gestational hypertension, adjustment for hospital eliminated relatively large elevations in odds ratios for PM_2.5_ and to a lesser extent NO_2_. Only the associations between air pollution and preeclampsia (including subsets of mild and severe preeclampsia) were minimally affected by adjustment for hospital. Adjusting for hospital as a random rather than fixed effect yielded similar results (data not shown).

**Table 1:**
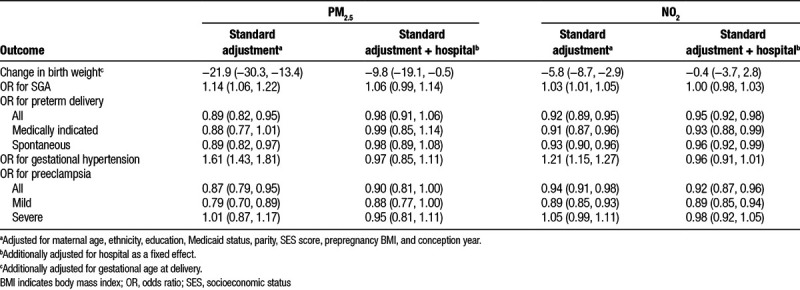
Estimated change in birth weight and odds of adverse pregnancy outcomes per 10 μg/m^3^ increase in PM_2.5_ and 10 ppb increase in NO_2_ comparing models with standard adjustment to those with standard adjustment plus hospital as a fixed effect

To understand the underlying relationships resulting in the impact of adjusting for delivery hospital, we first examined the association between delivery hospital and estimated residential air pollution levels of the women who delivered at that hospital. Maternal characteristics including neighborhood socioeconomic status were predictive of PM_2.5_ (*R*^2^ ranging from 0.22 to 0.31 across trimesters) and to a lesser extent NO_2_ (*R*^2^ from 0.12 to 0.22 across trimesters, Table 2). Delivery hospital had substantial additional predictive value for air pollution levels, increasing the model *R*^2^ for PM_2.5_ to 0.33–0.43 and for NO_2_ to 0.31–0.41. To quantify the magnitude of the association between delivery hospital and air pollution levels, we rank ordered hospitals by adjusted mean residential air pollution levels of study participants who delivered there and divided the distribution into quartiles (Table 3). Comparing each of the upper three quartiles to the lowest quartile, we found that the adjusted mean increase in PM_2.5_ (in units of μg/m^3^) across quartiles for second trimester exposure estimates was 0.57 (95% CI = 0.54, 0.59), 1.44 (95% CI = 1.42, 1.46), and 2.06 (95% CI = 2.04, 2.08) for the second, third, and fourth quartiles, respectively. Comparable figures for NO_2_ (in units of ppb) were 2.31 (95% CI = 2.25, 2.36), 4.40 (95% CI = 4.34, 4.46), and 6.46 (95% CI = 6.40, 6.51) across quartiles of second trimester exposure estimates. There was no pattern of association between hospital characteristics (city versus private ownership, level of care, number of obstetrical beds) and residential air pollution levels of the women who delivered there based on minimal increases in the *R*^2^ values (Table 2).

**Table 2:**
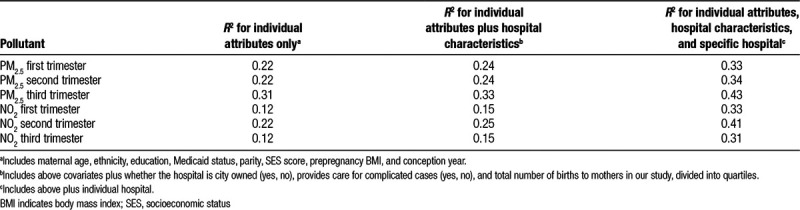
Proportion of variance in residential air pollution based on individual covariates, hospital attributes, and specific hospital

**Table 3:**
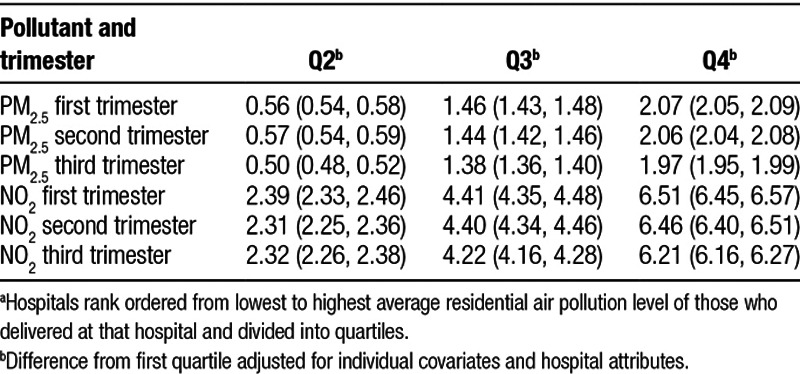
Estimated increment in air pollution levels (μg/m^3^ for PM_2.5_ and ppb for NO_2_) across quartiles for women who delivered at that hospital^a^

We next quantified the magnitude of association between delivery hospital and pregnancy outcome measures (Table 4). Women delivering at city-owned hospitals had a lower risk of medically indicated preterm delivery and a higher risk of spontaneous preterm delivery and being diagnosed with gestational hypertension or preeclampsia, even after adjustment for individual attributes, neighborhood socioeconomic status, and maternal residential air pollution levels. Hospitals that provide care for complicated cases tend to have births of lower average weight (and higher risk of SGA) and a higher likelihood of being diagnosed with gestational hypertension or severe preeclampsia. The number of obstetric beds in the hospital was positively associated with risk of medically indicated preterm delivery, gestational hypertension, and severe preeclampsia.

**Table 4:**
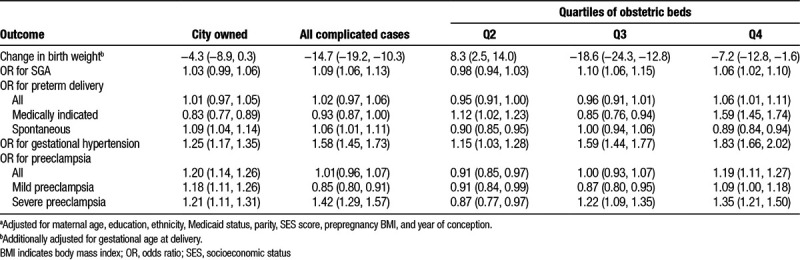
Change in term birth weight or odds ratio for adverse event by hospital strata for city-owned hospital, hospitals that accept all complicated cases, and across quartiles of obstetrics beds after adjustment for individual characteristics^a^

To quantify the strength of individual delivery hospitals as predictors of outcome, we again rank ordered hospitals from lowest to highest according to adjusted risk for each outcome (or mean birth weight) and divided them into quartiles (Table 5). Even after adjusting for individual characteristics and hospital attributes, delivery hospital was strongly predictive of all outcomes, with mean birth weight increased by 77 g in the fourth versus the first quartile, and the adjusted odds ratios for pregnancy complications ranged from 1.5 to 5.8 in the fourth versus first quartile. Comparing Tables 4 and 5, the overall influence of individual hospitals on pregnancy outcome measures is greater than reflected in the association of the specific hospital attributes we were able to consider.

**Table 5:**
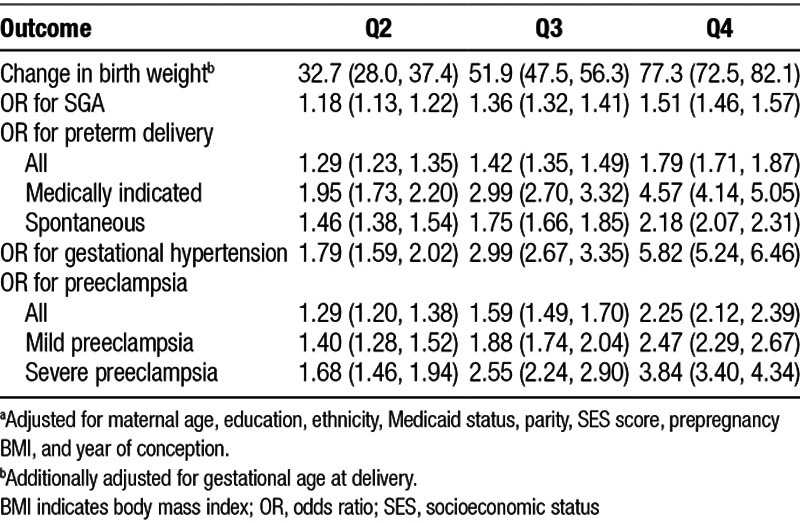
Change in term birth weight or odds ratio for adverse event by quartiles of delivery hospital rank ordered from lowest to highest risk of adverse outcome after adjustment for individual characteristics^a^

### Interpretation of results through directed acyclic graphs

**Figure 1.**
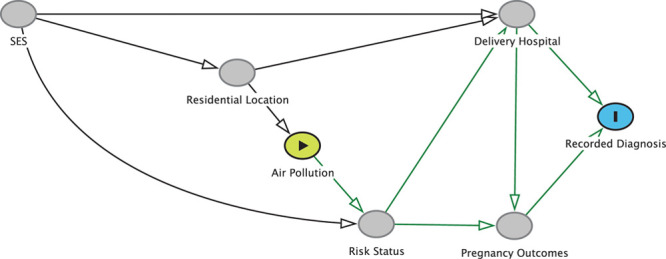
Directed acyclic graph depicting a study of the effects of ambient air pollution on birth outcomes. Long-term exposure to air pollution during pregnancy is largely influenced by location of the maternal residence. Delivery hospital is both a descendent of potential confounders between air pollution and birth outcomes and a potential causal intermediate. Adjustment for delivery hospital may reduce bias from confounding but also introduce bias by conditioning on a collider and causal intermediate.

To make explicit the considerations bearing on the risks and benefits of adjusting for delivery hospital, we developed a DAG (Figure 1) that includes air pollution and birth outcome—which could be birth weight or gestational age at delivery—as well as delivery hospital, socioeconomic status, residential location, and risk status, which refers to the presence of complications such as hypertensive disorders that are known before delivery. Note that this scenario only applies to spatially determined residential exposure, not temporal influences on ambient air pollution levels that are presumably unrelated to delivery hospital.

Socioeconomic status (SES) is well recognized as a strong determinant of residential location, and for this model, residential location is the sole determinant of air pollution exposure. Residential location, in turn, influences selection of delivery hospital in conjunction with SES and correlates of SES such as source of medical care and insurance coverage. There is some evidence that air pollution can influence risk of hypertensive disorders and perhaps other markers of risk status, and risk status is a strong predictor of pregnancy outcome and having an influence on the selection of the delivery hospital. Finally, the delivery hospitals may have different practices with regard to interventional delivery directly affecting the gestational age at delivery and indirectly affecting the birth weight distribution by gestational age. Interventions are often based on a combination of gestational age and estimated fetal weight to assess growth restriction and such interventions could alter the birth weight for gestational age distributions. In addition, delivery hospitals may differ in their completeness or accuracy of coding certain pregnancy complications such as preeclampsia, effectively creating an association between delivery hospital and risk of a diagnosed complication.

Thus, in Figure 1, we have three pathways involving delivery hospital with different implications for adjustment. First, to the extent that air pollution influences risk status and risk status influences choice of delivery hospital, delivery hospital is a causal intermediate between pregnancy risk and outcomes and should not be adjusted because doing so would block some of the effect of air pollution that is mediated by influences on the risk status of the pregnancy. In this scenario, adjustment for delivery hospital has the potential to introduce bias in the measured association between air pollution and pregnancy outcome. In addition, if there are any unmeasured common causes of pregnancy risk or outcomes and choice of delivery hospital (not shown in the Figure 1), then adjustment for delivery hospital could induce bias through conditioning on a collider.

On the other hand, residential location influences both air pollution levels and choice of delivery hospital, which in turn independently influences pregnancy outcomes. This reflects an aspect of spatial location that influences outcome separate from SES or other spatial correlates, namely through choice of delivery hospital. This pathway would make delivery hospital a confounder that should be adjusted to isolate the effect of air pollution on pregnancy outcome and failure to adjust would produce bias in the measured association between air pollution and pregnancy outcome. In addition, pregnancy outcomes as noted in administrative or medical records are inevitably misclassified to some degree and the magnitude of that outcome misclassification may plausibly vary across delivery hospitals. Moreover, the importance of hospital as a confounder of either actual or measured pregnancy outcomes can vary across air pollutants, potentially complicating the interpretation of the apparent relative importance of multiple pollutants examined in an analysis.

Thus, we propose that delivery hospital is simultaneously a marker of potential confounding by residential location, a marker of differential outcome misclassification, and potentially a causal intermediate on the pathway from air pollution to pregnancy risk to adverse birth outcomes. If true, adjustment for or stratification by delivery hospital would be expected to reduce bias from confounding or impact of measurement error, but also potentially induce bias by inappropriately adjusting for a causal intermediate or conditioning on a collider.

## Discussion

We and others have previously demonstrated associations between estimates of maternal residential air pollution levels and reduced fetal growth and higher risk of adverse pregnancy outcomes. However, most prior studies have not considered whether to adjust for delivery hospital as a potential confounder. We found evidence that adjustment for delivery hospital (either as a fixed or random effect) had a meaningful impact on many of the estimated associations between air pollution and pregnancy outcome, with plausible reasons that delivery hospital could be acting as a true confounder but also reasons for concern that adjustment for hospital could introduce bias by either conditioning on a potential causal intermediate or inadvertently opening a noncausal pathway between residence and pregnancy outcome. Adjustment for delivery hospital shifted observed associations toward the null, particularly for odds of gestational hypertension and markers of fetal growth, but that does not indicate whether the hospital-adjusted or hospital-unadjusted estimate is a more valid indicator of the causal effect of air pollution on outcome.

In choosing between adjusting and not adjusting for delivery hospital, a judgment must be made regarding which is more likely to be the largest source of bias. Extrapolating from knowledge of the underlying associations, the scenario that calls for adjustment will often be more compelling. It is clear that residential location in this study and many others is the sole determinant of air pollution levels and is very likely to independently influence choice of delivery hospital. The scenario in which bias is introduced by adjustment for delivery hospital depends on air pollution influencing risk status in recognized ways which in turn affects choice of delivery hospital. Although there is some evidence that air pollution may affect mediators of pregnancy outcome, effect sizes appear to be small at least for hypertensive disorders^1^ and are speculative for other features of risk status calling into question the quantitative impact of inappropriate adjustment for such factors.

An option to control for the residual spatial confounding of location that may result from the correlation of location and delivery hospital would be to adjust for a marker of residential location that is sufficiently refined to capture the predictiveness for delivery hospital but not so small as to result in nearly homogeneous air pollution levels (i.e., overadjustment). For example, in the United States, one might condition on ZIP Code or an aggregation of Census Tracts as in a recent analysis in this population.^16^ By not directly adjusting for the specific hospital, the risk of introducing bias from conditioning on an intermediate is eliminated, while adjusting for location in a manner that predicts delivery hospital helps minimize or eliminate residual confounding through delivery hospital.

There is evidence from previous studies bearing on a number of the arrows reflected in the DAG. Associations of air pollution with fetal growth and gestational hypertension were most strongly affected by adjustment for hospital, implying that those conditions are more susceptible to inter-hospital differences. While lacking definitive information, it might be hypothesized that gestational age-adjusted fetal growth is a measure of whether the pregnancy has been allowed to continue or has been ended with interventional delivery, although that would lead to the expectation that medically indicated preterm birth would likewise be strongly influenced by adjustment for hospital. An earlier report of a higher proportion of medically induced compared with spontaneous deliveries in a teaching versus community hospital supports the notion that hospital can influence delivery outcome in a systematic way.^17^

Gestational hypertension is often mild and subject to varying completeness of reporting, likely to be more variably reported than preeclampsia as was found in a Danish study,^18^ with severe preeclampsia more accurately reported than mild preeclampsia in a US population.^19^ This may account, in part, for the greater impact of hospital adjustment on gestational hypertension than preeclampsia in our data. In addition, the risk status of the pregnancy, often predictable well before delivery, can have bearing on the choice of delivery hospitals. Such a pattern of referrals would still provide the link between delivery hospital and pregnancy outcome, and because the conditions that defined “high risk” precede the outcome measures (in this case, “high risk of being diagnosed”), this mechanism would also constitute a valid basis for confounding.

The relationship between delivery hospital and pregnancy outcome is likely to be present across most geographic areas and at varying spatial scales, at least in the United States, because pregnancy risk status, proclivity to intervene, and coding of conditions are subject to variation across settings. Perhaps in settings or countries in which medical care is more standardized, delivery hospital would have less potential as a confounder than in the United States where the latitude for variation in clinical protocols and coding practices is substantial.

Variation in exposure across hospitals in relation to those influences on outcome may be random, and the complex spatial organization of residential locations, hospitals and their referral patterns, and air pollution levels is likely to vary across settings. In New York City, a number of the largest hospitals are located in Manhattan, in densely populated urban areas with relatively high pollution. Although other geographic areas may well have a different spatial profile of residential air pollution and delivery hospital, some nonrandom pattern of association may be present. What the detailed analysis of the relationship between hospital and air pollution revealed is that it is not primarily a function of hospital characteristics (e.g., ownership, size) but associated with the individual hospital. This may not be the same across different pollutants, as was reflected in the varying impact of adjustment on risk estimates for PM_2.5_ and NO_2._

In addition to the concern with generalizability, we were limited in our ability to examine the underlying determinants of the patterns that were observed. Although we can speculate about the ways in which hospitals differ in clinical and coding practices, for example, we do not have empirical data to confirm or refute the hypothesis that these are the causes of the association observed. Similarly, the reason hospitals vary in the residential exposure profile of the women who deliver there is presumed to be residential location, but we did not undertake a detailed analysis to examine the pattern of residence among women delivery at each specific hospital.

For these reasons, we offer this observation as a stimulus to encourage others who have examined air pollution (or other spatial factors) and pregnancy outcome to consider the role of delivery hospital more carefully and consider adjusting for some proxy of hospital catchment area. This would apply not only to studies of all the hospitals serving residents of a defined geographic area but also to studies based at a single hospital, where exposure variation will be constrained by the catchment area, but clinical care and coding of outcomes would presumably be homogeneous. Although we cannot offer definitive guidance, our findings encourage others to ask the question of whether delivery hospital is influencing their results given the often subtle effects of air pollution that are nonetheless of public health concern. When delivery hospital is found to be associated with residential air pollution, which will often be the case, and independently with the health outcomes, further empirical analysis would be warranted to more fully consider the possibility of confounding.

## Conflicts of interest statement

The authors declare that they have no conflicts of interest with regard to the content of this report.

This work was supported by Grants R01-ES019955 and R21-ES023073 from the National Institute of Environmental Health Sciences.

The data used for this analysis is derived from resources of the New York City Department of Health and Mental Hygiene and the New York State Department of Health, Statewide Planning and Research Cooperative System. To obtain the data and replicate these findings, investigators would be required to seek a collaboration with those agencies.

## Acknowledgments

The authors would like to thank Drs. Thomas Matte, Francesca Dominici, Jane Clougherty, and Jennifer Bobb, and Ms. Beth Elston for their notable contribution in developing and implementing the study on which these results are based.
